# Comparison of the effects of three different (-)-hydroxycitric acid preparations on food intake in rats: response

**DOI:** 10.1186/1743-7075-3-26

**Published:** 2006-07-17

**Authors:** Harry G Preuss, Manashi Bagchi, Debasis Bagchi

**Affiliations:** 1 Department of Physiology and Biophysics, Georgetown University Medical Center, Washington, DC 20057, USA; 2 InterHealth Research Center, Benicia, CA 94510, USA; 3 Department of Pharmacy Sciences, Creighton University Medical Center, Omaha, NE 68178, USA

## Abstract

A response to Louter-van de Haar J, Wielinga PY, Scheurink AJ, Nieuwenhuizen AG: Comparison of the effects of three different (-)-hydroxycitric acid preparations on food intake in rats. *Nutr Metabol *2005, 2:23

## Text

Louter-van de Haar J, et al. compared the effects of supplementation of three commercially available (-)-hydroxycitric acid (HCA) preparations [Regulator, Citrin K and Super CitriMax] on food intake and body weight. Two doses (150 mg HCA/kg and 300 mg HCA/kg) were used for each preparation over a period of four days. There are significant flaws in this study, which we have outlined below.

Two series of experiments were conducted. The first series of experiments determined the effect of a single dose of each preparation on food and water intake for the following 46 hrs in a four-day placebo-controlled cross-over experiment. In the second series of experiments, HCA preparations were given twice a day for four days to study the effects of repeated doses of the component on food and water intake.

The authors concluded that Regulator and Citrin K were shown to be potent inhibitors of food intake in rats, whereas Super CitriMax showed only small and more inconsistent effects.

There are several major weaknesses in this study, not the least of which were the small number of animals used in the study (n = 6 per group) and the short duration of the experiment. It is also unclear what the sequencing was of the supplements. It said that the animals were administered either placebo or a treatment then were crossed-over, but does not disclose which treatment groups were administered before or after the placebo. For example, if the Super CitriMax was administered before the placebo, its appetite suppression effects may have been carried over to the placebo portion of the experiment, minimizing any differences; while much greater differences may have been observed if the other treatments followed placebo administration. A 46-hour washout period was used, however, there is no data to support this as being a sufficient amount of time to clear the system of the effects of HCA and, in fact, Super CitriMax's ability to boost serotonin levels and reduce appetite may have carried over for several days, resulting in reduced food intake in the placebo group.

Furthermore, at the higher dosage levels where Regulator and Citrin K were reported superior to Super CitriMax, the food intake at the end of 24 hours of animals taking Super CitriMax was actually less than that of animals taking either Regulator or Citrin K (20.0 versus 20.9 and 23.0, respectively); however, for some reason the animals taking the placebo in the Super CitriMax group consumed significantly less food than the animals consuming either Regulator or Citrin K (19.6 versus 24.8 and 25.6, respectively), thus the difference between the Super CitriMax treatment and placebo groups was not as great. Was Super CitriMax the only group to be given the treatment first followed by the placebo?

Also, while the authors call four days a "long-term study," this is simply an inadequate period of time to demonstrate consistent appetite suppression and weight loss using HCA. HCA is a supplement, not a drug. Previous studies using Super CitriMax, both in animals and humans have demonstrated significant appetite suppression and weight loss over a greater span of time (six to eight weeks) [1-3].

The authors state that the high calcium and low potassium content of CitriMax (a calcium salt of HCA; 70% soluble in water) may affect its solubility and bioavailability. However, the authors are confusing CitriMax with Super CitriMax, the latter being a novel calcium/potassium salt of HCA that is highly water soluble (100%) and, in contrast to Regulator and Citrin-K, has been proven bioavailable in humans [4]. In addition, calcium offers the added benefit of increasing lipid metabolism, resulting in lipolysis and preserving thermogenesis during caloric restriction, accelerating weight loss [5,6].

The authors further suggest that Super CitriMax may consist of HCA lactone, a less effective form of HCA. However, HCA lactone analysis of Super CitriMax will show that its lactone content is less than 1%.

It should also be noted that several independent studies from multiple universities have shown that Super CitriMax modulates important neurotransmitters (serotonin) and neuropeptides (neuropeptide Y) involved in appetite control [6-8], something that Regulator and Citrin K have not shown. Clinical studies on Super CitriMax have demonstrated significant gradual reduction of appetite as measured by remaining food over a period of eight weeks [1,2], while no such human studies have been published on Citrin K and Regulator. In any event, the present study is an inadequate model for comparing HCA efficacy, and the speculation the authors offer on Super CitriMax is poorly informed.

## References

[B1] Preuss HG, Bagchi D, Bagchi M, Sanyasi Rao CV, Satyanarayana S, Dey DK (2004). Efficacy of a novel, natural extract of (-)-hydroxycitric acid (HCA-SX) and a combination of HCA-SX, niacin-bound chromium and *Gymnema sylvestre *extract in weight management in human volunteers. Nutr Res.

[B2] Preuss HG, Bagchi D, Bagchi M, Sanyasi Rao CV, Dey DK, Satyanarayana S (2004). Effects of a natural extract of (-)-hydroxycitric acid (HCA-SX) and a combination of HCA-SX plus niacin-bound chromium and *Gymnema sylvestre *extract on weight loss. Diabetes Obes Metab.

[B3] Mattes RD, Bormann L (2000). Effects of (-)-hydroxycitric acid on appetite variables. Physiol Behav.

[B4] Loe YC, Bergeron N, Rodriguez N, Schwarz JM (2001). Gas chromatography/mass spectrometry method to quantify blood hydroxycitrate concentration. Anal Biochem.

[B5] Leonhardt M, Hrupka B, Langhans W (2001). Effect of hydroxycitrate on food intake and body weight regain after a period of restrictive feeding in male rats. Physiol Behav.

[B6] Downs BW, Bagchi M, Subbaraju GV, Shara MA, Preuss HG, Bagchi D (2005). Bioefficacy of a novel calcium-potassium salt of (-)-hydroxycitric acid. Mutation Res.

[B7] Ohia SE, Opere CA, LeDay AM, Bagchi M, Bagchi D, Stohs SJ (2002). Safety and mechanism of appetite suppression by a novel hydroxycitric acid extract (HCA-SX). Mol Cell Biochem.

[B8] Roy S, Rink C, Khanna S, Phillips C, Bagchi D, Bagchi M, Sen CK (2004). Body weight and abdominal fat gene expression profile in response to a novel hydroxycitric acid based dietary supplement. Gene Expression.

